# Special Issue of “Material Analysis in Cultural Heritage”

**DOI:** 10.3390/ma16062370

**Published:** 2023-03-16

**Authors:** Žiga Šmit, Eva Menart

**Affiliations:** 1 Faculty of Mathematics and Physics, University of Ljubljana, SI-1000 Ljubljana, Slovenia; 2 Jožef Stefan Institute, SI-1000 Ljubljana, Slovenia; 3 National Museum of Slovenia, Prešernova 20, SI-1000 Ljubljana, Slovenia

The objects of cultural heritage represent memories of human activities from the past. Therefore, we pay special attention to their preservation, and study them to learn more about the lives and creativity of our ancestors. The present book offers insight into several analytical techniques applied to selected heritage materials.

Glass is now a leading technological material, especially in the field of optical communications. It was also useful and widespread in the past. In Roman times and late antiquity, the production of glass attained industrial dimensions. Raw glass was produced in Egypt and Palestine, exploiting local siliceous sands and alkalis from dry Egyptian lakes as raw materials. According to the glass composition, several glass types were recognized in an early study [1], which is still valid today [2]. A review paper by Balvanović et al. [3] studies the development and distribution of glass types of Foy Serie 3.2 and Foy Serie 2.1 in the Mediterranean during late antiquity. Glass from the 1st to 4th century from Histria and Tomis, in present-day Romania, was studied through prompt-gamma activation analysis by Bugoi et al. [4], showing both early Roman and later 3.2 and 2.1 glass types. 

Ceramic objects are often covered in glazes to improve their visual impression and increase their stability and durability. The composition of glazes used on ancient Chinese screen walls was studied by scanning electron microscopy (SEM) and X-ray diffraction (XRD), and the degradation processes were studied from a conservation point of view by Jingyi Shen et al. [5]. In goldwork masterpieces from the Quing Dynasty, it was found that the cobalt pigment in blue enamel was likely imported from Europe, as demonstrated by a non-destructive investigation through optical microscopy, Raman microspectroscopy, and X-ray microfluorescence spectroscopy by Colomban et al. [6]. For porcelain, it is important to show whether the products were made in a renowned or local workshop. Specifically in painted porcelain, an efficient analytical tool for surface and bulk analysis was found in position-sensitive X-ray fluorescence spectrometry (XRF) and prompt-gamma activation analysis (PGAA) by Szentmiklósi et al. [7]. The preservation of metals is another difficult task for conservation science, as they are prone to corrosion. One of the most challenging problems is the conservation of iron artifacts retrieved from a marine environment, which is the topic of the paper by Minghao Jia et al. [8]. Two papers deal with masonry and architectural remains. Fragata et al. [9] describe bricks and mortars in the Roman city of Bracara Augusta, modern-day Braga in Portugal. Chemical/elemental analysis performed by XRF demonstrates that the composition of the bricks differs according to their functionality, depending on whether they are being used as coatings, floorings, or masonry mortars, and their age of origin between the 4th and 7th centuries. In modern buildings, produced mainly by reinforced concrete since the middle of the 20th century, the deterioration and strength of the concrete need to be constantly monitored. Non-destructive tests with ultrasound are generally preferred, but their reliability needs to be regularly checked by destructive tests. Vona [10] constructed a comprehensive database to assess the reliability of the relationship between destructive and non-destructive methods for in situ concrete testing. 

Studies of lithic materials conclude with a thrilling story of “magic” black stones that have been preserved in the childhood home of famous Italian poet Giacomo Leopardi. The analysis performed by Santi et al. [11] suggests that the stones originate from Tuscany and were used by the Romans as counterweights. However, according to popular belief, they could also have been used as weights for torturing early Christians. Modern analytical techniques are further useful tools for the characterization of paint pigments. During the 19th and 20th centuries, yellow pigments based on cadmium sulfide (CdS) attained popular use. However, the pigment is subject to degradation with time. A study by Pisu et al. [12] employed the methods of SEM-EDS (energy-dispersive spectroscopy), Raman, and XRD on model samples painted with CdS, demonstrating its degradation into cadmium sulfate. 

Another important aspect of culture, past or present, is music. Stanciu et al. studied the art of making ancient violins [13]. The authors investigated the structure and density of wood. They performed X-ray imaging and computed tomography on several violins manufactured by famous violin makers. 
